# Restrictive Versus Standard Intravenous Fluid Therapy and Endothelial Glycocalyx Shedding in ICU Patients With Septic Shock—A Preplanned Sub‐Study of the Randomized CLASSIC Trial

**DOI:** 10.1111/aas.70156

**Published:** 2025-12-01

**Authors:** Jens Christensen, Praleene Sivapalan, Tine Sylvest Meyhoff, Hans Järnbert‐Pettersson, Anders Perner, Morten Hylander Møller, Theis Lange, Peter Buhl Hjortrup, Eva Joelsson‐Alm, Sandra Jonmarker, Fredric Sjöberg, Johan Mårtensson, Anna Håkansson Gladh, Maria Cronhjort

**Affiliations:** ^1^ Department of Clinical Science and Education Södersjukhuset, Karolinska Institutet Stockholm Sweden; ^2^ Copenhagen University Hospital–Rigshospitalet Copenhagen Denmark; ^3^ University of Copenhagen, Section of Biostatistics Copenhagen Denmark; ^4^ Department of Physiology and Pharmacology Section of Anaesthesia and Intensive Care, Karolinska Institutet Stockholm Sweden

## Abstract

Shedding of the endothelial glycocalyx may be a pathophysiological mechanism in septic shock, to which intravenous (IV) fluid therapy may contribute. We aimed to investigate the effects of restrictive versus standard IV fluid therapy on glycocalyx shedding in adult intensive care unit (ICU) patients with septic shock. In this preplanned sub‐study of the CLASSIC trial, ICU patients with septic shock were randomized to restrictive or standard IV fluid therapy at one Danish and one Swedish ICU between February 2020 and October 2021. Plasma markers of glycocalyx shedding were measured at four timepoints: within the first hour after randomization (T0), the following morning (T1), the morning after (T2), at ICU discharge or up to 90 days after randomization (T3). The primary outcome was the change in plasma hyaluronan levels from T0 to T1. A total of 54 patients were included, below the planned sample size of 200, leading to important differences between groups. Mean hyaluronan levels decreased by 11 ng/mL (95% CI 35–41) more in the restrictive group compared to the standard group from T0 to T1. The interaction effect between group and time was non‐significant (*p* value: 0.872). In this underpowered sub‐study we found no statistically significant difference in endothelial glycocalyx shedding between adult ICU patients with septic shock randomized to restrictive versus standard IV fluid therapy. We consider these findings hypothesis‐generating; further research is needed to confirm these results.

**Editorial Comment:**

In this substudy of the CLASSIC trial, glycocalyx degredation products and related substances were measured serially in sepsic study participants randomized to restrictive or usual fluid treatment protocols. No differences between treatment glycocalyx degredation product and related substance levels were observed.

**Trial Registration:** ClinicalTrials.gov identifier: NCT04282252.

**Abbreviations**
Ang‐2: angiopoietin‐2
CI: confidence interval
CLASSIC: conservative versus liberal approach to fluid therapy of septic shock in intensive care
GAG: glycosaminoglycan
GI: gastrointestinal
ICU: intensive care unit
IL‐6: interleukin‐6
IQR: inter quartile range
IV: intravenous fluid
MRproADM: mid regional pro adrenomedullin
SMS‐ICU: Simplified Mortality Score for the Intensive Care Unit
TNFR1: tumor necrosis factor receptor 1

## 1 Introduction

Septic shock is a common and life‐threatening condition. Although much is still unknown regarding the pathophysiology, one of the accepted characteristics of this syndrome is endothelial glycocalyx shedding [1, 2]. While our understanding of the consequences is still limited, endothelial glycocalyx shedding during septic shock may contribute to hypovolemia, oedema, vasodilation, activation of coagulation, and dysregulation of the inflammatory response, all key clinical features of septic shock [2, 3, 4, 5]. Therefore, a better understanding of the mechanisms behind this endothelial shedding, and potential ways to prevent it, could be of clinical value.

The glycocalyx is a gel‐like semipermeable meshwork coating the luminal surface of all healthy blood vessels throughout the body, with a pivotal role in vascular physiology and pathology [6, 7]. This carbohydrate‐rich structure is mainly synthesized by endothelial cells and consists of glycosaminoglycans (GAGs), glycoproteins, proteoglycans and adherent plasma proteins. In addition to septic shock, the glycocalyx is degraded in various other and often overlapping clinical situations, including exposure to endotoxins [8], infection [9] and critical illness [10]. The degree of glycocalyx shedding can be estimated by the increase of circulating plasma levels of its constituents such as the GAGs hyaluronan and syndecan‐1, the glycoprotein CD‐44 as well as the signaling substances angiopoietin‐2 (Ang‐2), interleukin‐6 (IL‐6), mid regional pro adrenomedullin (MRproADM) and tumor necrosis factor receptor 1 (TNFR1) [11, 12, 13, 14].

Intravenous (IV) fluid bolus therapy also has the potential to cause glycocalyx shedding [15, 16, 17, 18]. Given that current fluid resuscitation guidelines in the treatment of septic shock recommend early administration of relatively large IV fluid boluses, it is plausible that this treatment could further aggravate the preexisting vascular damage [19]. Recent studies of septic patients have shown an association between the volume of administered IV fluid and plasma markers of glycocalyx shedding, suggesting that fluid load could induce iatrogenic endothelial injury in this patient group [9, 20]. Yet, it is uncertain to what extent fluid therapy contributes to the shedding of the glycocalyx in patients with septic shock.

In the international randomized clinical trial Conservative versus Liberal Approach to Fluid Therapy of Septic Shock in Intensive Care (CLASSIC), adult patients with septic shock were randomized to restrictive versus standard IV fluid therapy [21]. In this preplanned sub‐study of the CLASSIC trial, we aimed to assess endothelial biomarkers in enrolled participants to investigate the effect of restrictive versus standard IV fluid therapy on glycocalyx shedding. We hypothesized that restrictive IV fluid therapy may result in less glycocalyx shedding, compared to standard IV fluid therapy, measured by the difference in change of hyaluronan between the two groups during initial intensive care unit (ICU) stay.

## 2 Method

### 2.1 Trial Design

This was a preplanned prospective sub‐study of the CLASSIC trial (NCT04282252). The CLASSIC trial was a European, parallel group, open label, multicenter, randomized clinical trial in which 1554 ICU patients with septic shock were randomized to receive restrictive versus standard IV fluid therapy. A detailed analysis plan for this sub‐study was published prior to data analysis [22]. This analysis plan differed from the initial ClinicalTrial.gov registration due to a subsequent decision to divide the original study into two separate studies.

### 2.2 Participants

Patients eligible for this sub‐study were those included in the CLASSIC trial at Södersjukhuset, Stockholm, Sweden and Rigshospitalet, Copenhagen, Denmark, from February 2nd, 2020, to October 22nd, 2021. The CLASSIC trial included adult ICU patients with septic shock according to the Sepsis‐3 criteria [23] who had received at least 1 L of IV fluid in the 24 h before screening. Patients were excluded if they had septic shock for more than 12 h, if they had life‐threatening bleeding, or acute burn injury > 10% of the body surface area, if they were pregnant or if consent could not be obtained [21, 24]. Patients provided consent to participate in both the CLASSIC trial and the sub‐study. Inclusion in the sub‐study depended on the availability of personnel on site to draw and handle blood samples.

### 2.3 Interventions

Patients were randomized to receive restrictive or standard IV fluid therapy for a maximum of 90 days during ICU stay. In the restrictive fluid group, IV fluid administration was only permitted in case of either: (1) severe hypoperfusion according to prespecified criteria (2) a documented fluid loss, (3) dehydration or electrolyte deficiencies requiring correction or (4) necessity to ensure a daily intake of 1 L of fluids [24]. If any of these conditions were met, an IV fluid bolus of 250 to 500 mL crystalloid fluid could be administered. In the standard fluid group patients were to be treated according to the Surviving Sepsis Campaign recommendations [25], thus no upper limit for IV fluid administration was set [24].

### 2.4 Outcomes

Primary outcome of this study was the change in plasma levels of the glycocalyx shedding marker hyaluronan between the two intervention groups at timepoints T0 to T1 as defined below. This time interval was chosen based on the Surviving Sepsis Campaign guidelines, which recommend early fluid resuscitation [25], as well as to avoid multiple testing.

Exploratory outcomes were the change of plasma levels of glycocalyx shedding markers hyaluronan, syndecan‐1, Ang‐2, CD44, TNFR1, MRProADM, and IL‐6 between the two intervention groups at timepoints T0 to T1, T2, and T3 as defined below.

### 2.5 Blood Sampling and Analysis

Blood samples were collected from patients at four different timepoints: first hour after randomisation in the CLASSIC trial (T0), in the morning the following day (T1), in the morning the day after (T2) and at ICU discharge/90 days (T3). Two blood samples were obtained at each timepoint, one 5 mL EDTA tube and one 4 mL Citrate tube. Samples could be taken either arterially or venously, from existing catheters or by needle puncture of a blood vessel. The samples were centrifuged no later than 3 h after sampling. After centrifugation plasma was distributed into 5 smaller test tubes for each timepoint and stored in a research freezer at least at −70°C. The samples were later sent for further analysis at a central laboratory located at Södersjukhuset. All blood sampling and handling were done according to a prespecified protocol.

Concentrations of hyaluronan were analyzed using the Quantikine ELISA kit (R&D Systems, DHYALO). Concentrations of Ang‐2 were also analyzed by the ELISA kit (Thermo Fisher Scientific, KHC‐1641) as were concentrations of MRProADM (BioSite, KSJ‐GZJWBQ‐96). Concentrations of Syndecan‐1, CD44, TNFR1, and Il‐6 were analyzed using the hMagnetic Luminex Assay 7 pPlex (Bio‐Techne LXSAHM‐07). The analytical performance of the biomarker assays is summarized in Table S1, including intra‐assay coefficients of variation (CV) and limits of quantification (LOQ).

### 2.6 Sample Size

Sample size estimation was based on hyaluronan concentrations established in a previous comparable study [9]. From these a sample size calculation using a standard deviation of 29 ng/mL of hyaluronan concentration revealed a sample size of *n* = 200 to obtain 80% power to detect a group difference in change of 11.5 ng/L from T0 to T1 with *α* = 0.05. Notably, a change in the study design from multiple primary outcomes to a single primary outcome was implemented after registration on clinicaltrials.gov but prior to the publication of the analysis plan [22]. As a result, the final sample size calculation differs from that listed on clinicaltrials.gov, where a more conservative *α* = 0.025 yielded a sample size requirement of *n* = 248.

### 2.7 Randomization

Randomization in the CLASSIC trial was performed using a centralized computer‐generated allocation sequence that was stratified by trial site and occurrence of metastatic or hematologic cancer. Patients were randomly allocated in a 1:1 ratio, in permuted blocks of 6 or 8, to be treated with restrictive fluid therapy or standard fluid therapy. Treatment groups were not blinded for patients, treating clinicians or investigators, but were concealed from the data and safety monitoring committee and trial statisticians [21, 24].

### 2.8 Statistical Methods

To answer the main research question, the mean difference in change of the concentration of hyaluronan between the restrictive and standard fluid groups at timepoints T0 and T1 was compared using a mixed effect linear model. For each outcome, we (a) estimated mixed models with a random intercept and fixed effect for time (T0 and T1 for the primary analysis, discrete timepoints T0–T3 for the exploratory analyzes) and treatment group (restrictive fluid versus standard fluid group) to investigate differences between the groups. The covariance structures between timepoints were modeled with a first‐order autoregressive model (AR [22]). To assess whether the differences between the groups were equal at different timepoints, we (b) tested interaction between time and treatment group. Finally (c), to adjust for possible imbalances between groups regarding sex, focus of infection, and illness severity Simplified Mortality Score for the Intensive Care Unit (SMS‐ICU [26]), these factors were to be added one at a time as a fixed effect to the primary model (b) if imbalances between groups were suspected. If the results from the adjusted model differed in a clinically relevant way, these factors were to remain as a fixed effect in the main model. *p* value < 0.05 was regarded as statistically significant. All statistical analyses were performed using IBM SPSS Statistics 2024 except for the generation of Figure 3 and multiple imputation analysis, which was created through R version 4.3.2 (R Core Team, R Foundation for Statistical Computing).

### 2.9 Missing Data

All analyses were based on all available data, that is, individuals with partially missing data were included. Missing data was assumed to occur “Missing at Random” (MAR). The main analysis was complemented with worst‐ and best‐case analyses where worst case was defined as the 90th percentile value and best case was defined as the 10th percentile value in the group to which the patient had been randomized. If an important clinical difference in the worst‐ and best‐case analyses was found, multiple imputation was to be performed as a sensitivity analysis. Multiple imputation (R package mice) with default arguments was used, with the exception that the number of imputations was set to 50. Notably, imputation was conducted solely for the outcome variable hyaluronan included in model (b), using predictive mean matching as the imputation method. Following the completion of multiple imputation, the emmeans package was used to calculate estimated marginal means from the imputed datasets.

### 2.10 Large Language Models

The large language model ChatGPT (OpenAI, San Francisco, CA, USA) was used to assist with language refinement during the drafting of this manuscript.

## 3 Results

### 3.1 Patient Characteristics

**FIGURE 1:**
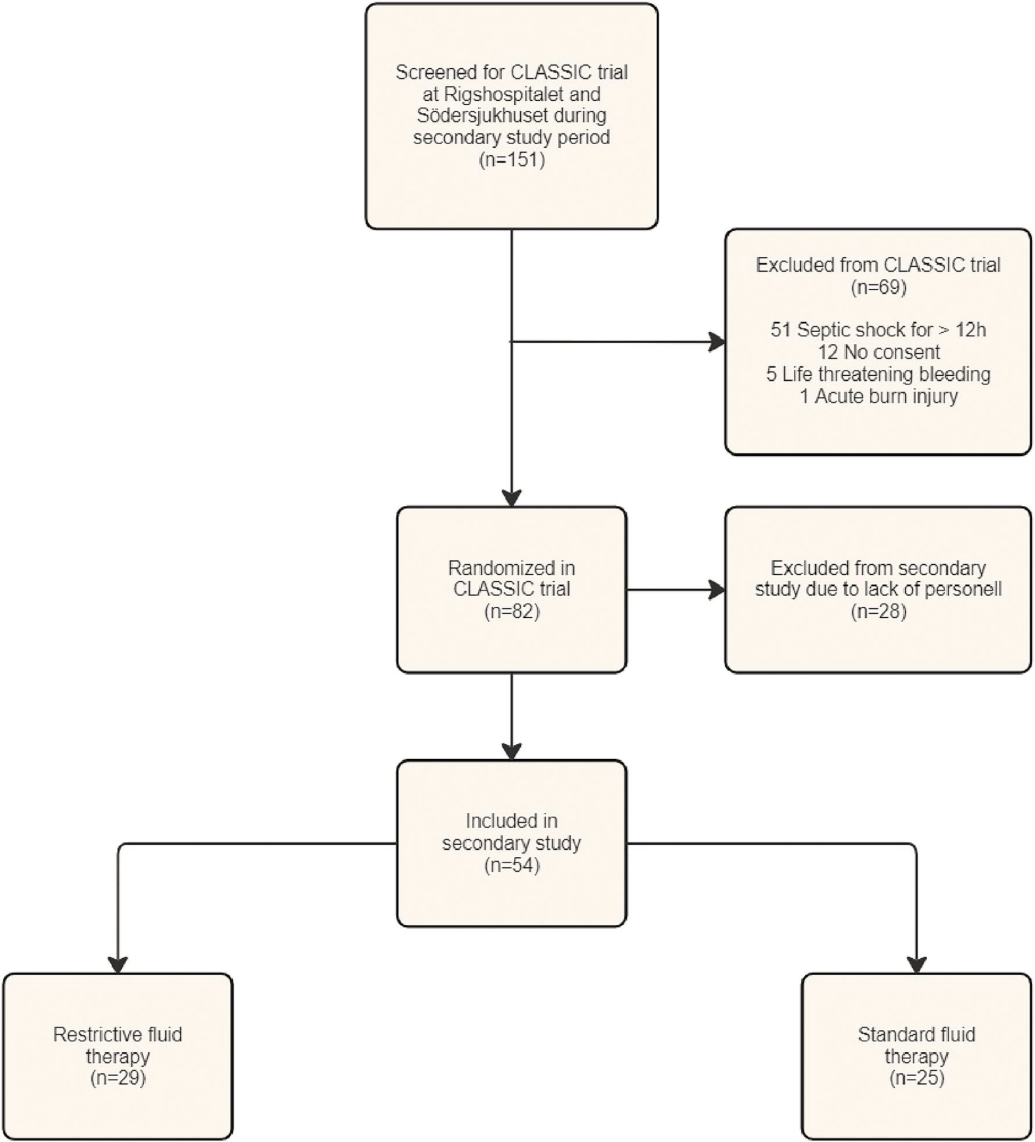
Screening, inclusion, and randomization.

Due to a lack of personnel during the COVID‐19 pandemic, inclusion in this sub‐study did not occur at the rate needed to achieve the preplanned sample size of 248 before the CLASSIC trial was completed. A total of 54 patients were included: 14 patients from Södersjukhuset and 40 patients from Rigshospitalet (Figure 1). A total of 29 patients were randomized to restrictive fluid therapy versus 25 patients to standard fluid therapy.

Baseline characteristics such as age, sex, median volume of IV fluid 24 h before randomization and predicted 90‐day mortality appeared similar between the two groups (Table 1). GI infection and use of respiratory support were more common in the restrictive fluid group compared to the standard fluid group (Table 1). Median time from T0 to T1 was 16.8 h in the restrictive group (IQR 10.8–21.0) compared to 18.8 h in the standard group (IQR 14.0–22.5).

**TABLE 1:** Patients characteristics at baseline.

Characteristics	Restrictive fluid (*N* = 29)	Standard fluid (*N* = 25)
Median age (IQR)—year	72 (63.5–77.5)	72 (70.0–78.0)
Male sex—no. (%)	18 (62.1)	16 (64.0)
Coexisting condition—no. (%)		
Hematologic or metastatic cancer	9 (31.0)	6 (24.0)
Ischemic heart disease or heart failure	3 (10.3)	9 (36.0)
Chronic hypertension	13 (44.8)	12 (48.0)
Long‐term dialysis^a^	1 (3.4)	3 (12.0)
Median time from ICU admission to randomization (IQR)—hour	3.5 (1.6–12.3)	4.8 (1.1–13.4)
Median predicted 90‐day mortality (IQR)—%^b^	23.0 (19.5–28.0)	24 (20.0–27.0)
Inclusion site—no. (%)		
Södersjukhuset IVA	6 (20.7)	8 (32.0)
Rigshospitalet	23 (79.3)	17 (68.0)
Source of ICU admission—no. (%)		
Emergency department or prehospital	9 (31.0)	5 (20.0)
Hospital ward	10 (34.5)	12 (48.0)
Operating or recovery room	9 (31.0)	7 (28.0)
Another ICU	1 (3.4)	1 (4.0)
Focus of infection—no. (%)^c^		
Gastrointestinal	10 (34.5)	5 (20.0)
Pulmonary	6 (20.7)	5 (20.0)
Urinary tract	4 (13.8)	8 (32.0)
Skin or soft tissue	5 (17.2)	3 (12.0)
Other	4 (13.8)	4 (16.0)
Body weight, blood values, and interventions		
Median body weight (IQR)—kg	79.0 (67.0–93.5)	78.0 (68.0–91.0)
Median highest plasma lactate (IQR)—mmol per liter^d^	3.5 (2.6–4.9)	3.9 (2.9–5.7)
Median highest dose of norepinephrine (IQR)—μg/kg/min^e^	0.28 (0.14–0.46)	0.25 (0.1–0.46)
Median volume of intravenous fluid 24 h before randomization (IQR)—ml^f^	2811 (1486–4032)	2983 (2050–3988)
Use of systemic glucocorticoid—no. (%)	11 (37.9)	6 (24.0)
Median highest plasma creatinine (IQR)—mg/dl^g^	147.0 (103.5–257.5)	154.0 (117.5–243.5)
Use of respiratory support—no. (%)^h^	13 (44.8)	8 (32.0)
Missing samples—no. (%)		
Any missing sample	25 (86.2)	18 (72.0)
Missing T0	14 (48.3)	4 (16.0)
Missing T1	2 (6.9)	0 (0)
Missing T2	5 (17.2)	8 (32.0)
Missing T3	20 (69.0)	14 (56.0)

*Note:* There was no missing baseline data.

Abbreviations: ICU, intensive care unit. IQR, interquartile range.

^a^ Long‐term dialysis was defined as the use of hemodialysis (or hemofiltration) or peritoneal dialysis at least once a week before hospital admission.

^b^ The predicted 90‐day mortality was calculated from the Simplified Mortality Score for the intensive care unit.

^c^ The listed location was the documented or suspected focus of infection at the time of randomization.

^d^ Shown are the highest plasma lactate levels within the 3 h before randomization.

^e^ The infusion rate of norepinephrine reflects the highest rate within the 3 h before randomization.

^f^ Volumes of intravenous fluid within the 24 h before randomization were defined as all crystalloid fluids, colloid fluids and blood products the patient had received within the 24 h before undergoing randomization, independent of location (in‐hospital or prehospital) and including intravenous fluids that contained medication or nutrition.

^g^ Values reflect the highest plasma creatinine level within the 24 h before randomization.

^h^ Respiratory support includes the continuous use of invasive or noninvasive mechanical ventilation or continuous positive airway pressure at baseline.

### 3.2 Fluid Characteristics and CLASSIC Outcomes

The restrictive fluid group received less IV fluid volume (defined as cumulative volumes of intravenous fluids administered in the ICU not including blood products and intravenous fluids with medication and nutrition) from randomization to timepoints 1 day, 5 days and at ICU discharge/90 days (Table 2). A similar pattern, although more pronounced, was seen for cumulative fluid balance at previously mentioned timepoints. Median total fluid volumes given (i.e., intravenous fluids, blood products, nutrition, intravenous and oral medications, and oral fluid intake) were also lower in the restrictive compared to the standard fluid group at timepoint 1 day. The opposite was seen for total fluid volumes at timepoints 5 days and ICU discharge/90 days, although the range at these timepoints was relatively wide (Table 2). In total, ≥ 1 IV fluid protocol violations were observed in 10 patients (34.4%) in the restrictive fluid group and 3 patients (12.0%) in the standard fluid group. The median number of days spent in ICU was 6 (IQR 4–10) in the restrictive fluid group and 4 (IQR 3–7) in the standard fluid group. Mortality at 90 days was 48.3% in the restrictive fluid group versus 28.0% in the standard fluid group. A detailed summary of fluid types is presented in Table S2.

**TABLE 2:** Cumulative fluid volumes and balances in milliliters.

	Restrictive fluid group (*N* = 29)	Standard fluid group (*N* = 25)	Difference (Restrictive vs. Standard)
Intravenous fluid volume^a^			
After 1 day^b^			
Median (IQR)	320 (0–940)	926 (215–1887)	−606
Mean	558	1213	−655
After 5 days			
Median (IQR)	1420 (471–2556)	2379 (1037–3555)	−959
Mean	2163	2941	−778
At ICU discharge^e^			
Median (IQR)	1795 (90–4377)	2715 (1037–5600)	−920
Mean	3399	3391	8
Total fluid volume^c^			
After 1 day^b^			
Median (IQR)	2163 (834–3382)	2361 (1514–3907)	−198
Mean	2180	2704	−524
After 5 days			
Median (IQR)	10,601 (6700–13,850)	8066 (5434–10,487)	2535
Mean	10,589	9654	935
At ICU discharge^e^			
Median (IQR)	13,644 (6701–29,250)	8066 (5440–20,823)	5578
Mean	25,835	14,113	11,722
Cumulative fluid balance^d^			
After 1 day^b^			
Median (IQR)	385 (41–1145)	890 (483–2364)	−505
Mean	321	1369	−1048
After 5 days			
Median (IQR)	646 (−1204–2679)	2045 (1209–4763)	−1399
Mean	682	2801	−2119
At ICU discharge^e^			
Median (IQR)	−215 (−1502–3246)	1323 (385–4763)	−1538
Mean	753	2386	−1633

*Note:* There was no missing fluid data.

Abbreviations: ICU, intensive care unit; IQR, interquartile range.

^a^ Cumulative volumes of intravenous fluids administered in the ICU (excluding blood products and intravenous fluids with medication and nutrition).

^b^ From the time of randomization to the next start of the 24‐h fluid chart used by the ICU.

^c^ Amounts represent the volumes of total fluid intake, including intravenous fluids, blood products, nutrition, intravenous and oral medications, and oral fluid intake.

^d^ Amounts represent the total volume of fluid intake minus the total fluid output, including urinary output, fluid removed by renal replacement therapy, and other fluid output (e.g., bleeding, ascites, diarrhea, or drain losses).

^e^ Up to 90 days.

### 3.3 Outcome

**FIGURE 2:**
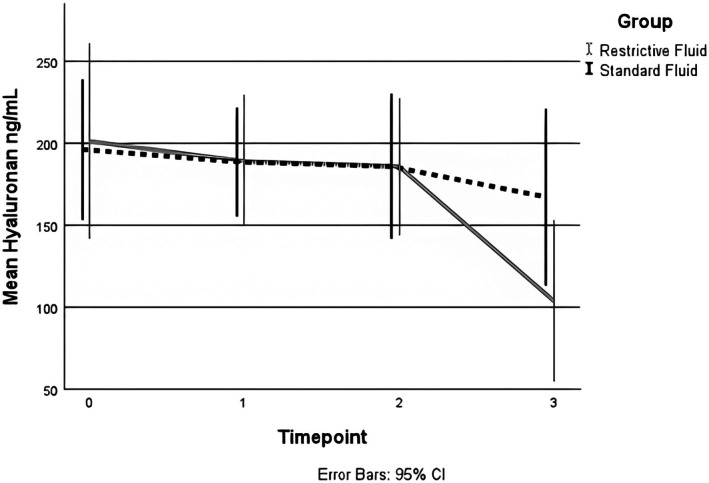
Graphical presentation of mean plasma levels of hyaluronan across all timepoints. Number of cases at each timepoint: T0: 36, T1: 52, T2: 41, T3: 20.

**FIGURE 3:**
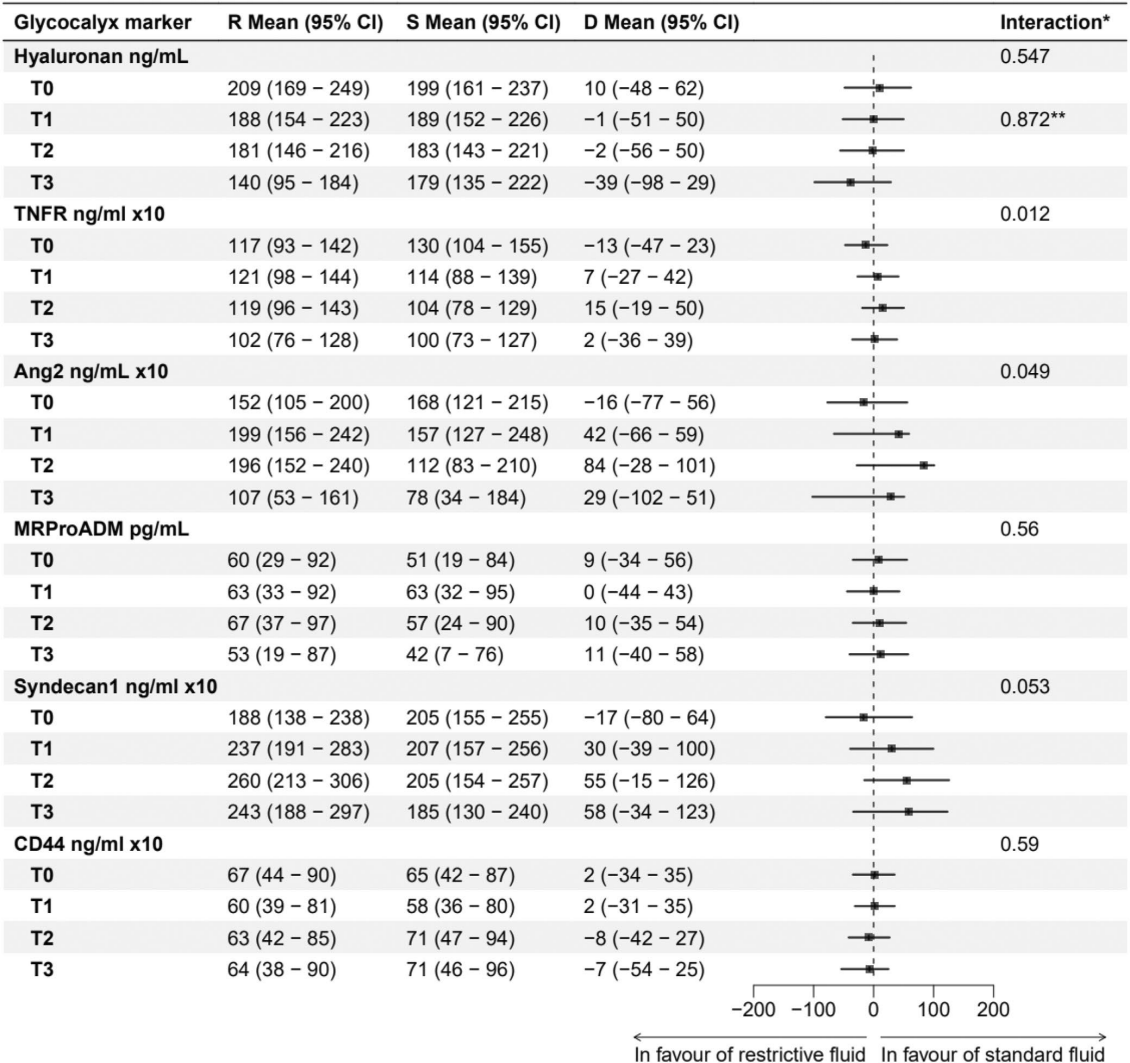
Forest plot. Forest plot showing differences in mean plasma levels of glycocalyx shedding markers between the restrictive group and standard fluid group, complemented with *p* values of interaction effects between group and time. R Mean: Mean plasma levels of glycocalyx shedding markers in the restrictive fluid group. S Mean: Mean plasma levels of glycocalyx shedding markers in the standard fluid group. D Mean: Differences in mean plasma levels of glycocalyx shedding markers between the restrictive and standard fluid group. *p values for interaction effects between group allocation and time across all timepoints. **p value for interaction effect between group allocation and time from timepoint T0 to T1.

Mean levels of hyaluronan decreased by 11 ng/mL (95% CI 35–41) more in the restrictive fluid group compared to the standard fluid group from T0 to T1. The difference between groups at T0 was 10 ng/mL (95% CI 48–62) (restrictive fluid group 209 ng/mL (95% CI 169–249), standard fluid group 199 ng/mL (95% CI 161–237)) and at T1 1 ng/mL (95% CI 51–50) (restrictive fluid group 188 ng/mL (95% CI 154–223), standard fluid group 189 ng/mL (95% CI 152–226)), (Figures 2 and 3). This change between the two groups was not statistically significant as the interaction between timepoint and group was non‐significant (*p* = 0.872, Figure 3).

Due to baseline imbalance between groups regarding focus of infection, a complementary post hoc analysis was performed with the addition of focus of infection to the primary model as a fixed effect. In this analysis mean levels of hyaluronan were reduced by 19 ng/mL (95% CI 13–51) in the restrictive fluid group from T0 (213 ng/mL (95% CI 172–253)) to T1 (194 ng/mL (95% CI 158–229)) compared to 16 ng/mL (95% CI 13–36) in the standard fluid group (T0 214 ng/mL (95% CI 175–253), T1 198 ng/mL (95% CI 160–235)), with a non‐significant interaction effect between timepoint and group allocation (*p* = 0.886). The results from this complementary analysis were not considered to differ from the primary analysis in a clinically significant way; therefore, we present the results without adjustment for focus of infection.

For exploratory outcomes, only TNFR1 and Ang‐2 showed significant group‐by‐time interactions (*p* values 0.012 and 0.049, respectively). In both cases, concentrations were lower in the restrictive group at T0 but higher at T1–3 compared to the standard fluid group (Figure 3).

### 3.4 Missing Values

A total of 67 missing values of primary and exploratory outcomes were observed in a total of 43 patients, constituting 31.0% of all values. These were distributed as follows: 18 missing values at T0, 2 missing values at T1, 13 missing values at T2, 34 missing values at T3 (Table 1). Opposing directions were seen in the best‐case analysis compared to the worst‐case analysis. In the best‐case analysis hyaluronan increased in both groups, whereas the worst‐case analysis showed a decrease in both groups (see Appendix A). Multiple imputations analysis displayed a reduction of mean hyaluronan values of 1 ng/mL (95% CI 73–70) more in the standard fluid group compared to the restrictive fluid group (see Appendix A).

## 4 Discussion

### 4.1 Main Findings

In this under‐powered preplanned sub‐study of the CLASSIC trial, we observed no statistically significant difference in the change of plasma hyaluronan levels during early fluid intervention among adult ICU patients with septic shock allocated to restrictive versus standard IV fluid therapy.

We did observe a statistically significant group‐by‐time interaction effect for exploratory outcomes TNFR1 and Ang‐2, suggesting that restrictive fluid therapy might have an impact on these biomarkers compared to standard fluid therapy. However, Ang‐2 and TNFR1 are also strongly correlated to the degree of inflammation [12, 27] and as we observed no statistically significant difference in the change of the other glycocalyx shedding biomarkers, this finding must be interpreted with caution.

### 4.2 Generalizability

Baseline values of hyaluronan in our study were similar to previous studies of critically ill and septic patients [9, 17, 28]. The observed change of hyaluronan over time in our study resembles changes described in septic patients in the emergency department, where peak concentrations were seen within the first 3 h upon arrival, followed by a decline [9, 28].

Previous publications have shown that IV fluid administration is associated with increased levels of glycocalyx shedding in non‐septic patients as well as in critically ill and septic patients without shock [9, 15, 16, 17, 18]. The fact that we found no such difference in ICU patients with septic shock could be because this population already displays a high degree of endothelial shedding upon ICU arrival, possibly partly due to the recommended initial fluid treatment prior to ICU admission as well as a higher disease burden. In the context of preexisting pronounced endothelial impairment, the glycocalyx shedding effect of further IV fluid resuscitation might be negligible. This theory is supported by a recent trial by Macdonald et al. in which 99 patients in the emergency department with suspected sepsis who had received > 1000 mL of IV crystalloid fluid were randomized to restrictive or standard fluid therapy, showing no statistically significant difference in glycocalyx shedding [28].

### 4.3 Strengths and Limitations

The study is strengthened by being a preplanned sub‐study to an international randomized clinical trial. Although the sample size was relatively small, a difference in administered IV fluid volume, total fluid volume and cumulative fluid balance between groups was achieved at T1, indicating adherence to the protocol. All laboratory analyses were performed at the same laboratory and investigators followed a preplanned sampling protocol, reducing the risks of local variations.

There are several limitations to our study. Group allocation was not blinded to staff or patients. Since the study period coincided with the COVID‐19 pandemic, research staff was scarce, resulting in a substantially lower final sample size than planned. Consequently, the study was underpowered to detect the prespecified effect size. A post hoc calculation indicated approximately 80% power to detect between‐group differences of about 22 ng/mL at *α* = 0.05, which underscores the imprecision reflected by the wide confidence intervals. A relatively large amount of missing data was noted, with predominance at T0 compared to T1. This is displayed by the opposing trends of hyaluronan change in the best‐case and worst‐case analysis, illustrating the uncertainty of the results. The results of the multiple imputations analysis illustrate the uncertainty of the results, as the small, nonsignificant difference between the groups disappeared. There were differences between groups. The most common focus of infection in the restrictive fluid group was GI infection compared to the standard fluid group where urinary tract infection was most common. Since GI infection generally is associated with larger fluid losses and more severe septic shock, this might have affected the volumes of fluids administered. The higher use of respiratory support in the restrictive fluid group as well as the higher 90‐day mortality in the restrictive fluid group could be further indication of this. This greater disease burden in the restrictive fluid group may also correlate with more extensive preexisting glycocalyx damage, an imbalance potentially masking beneficial effects of restrictive fluid therapy. Worth noting is that in the full population of the CLASSIC trial similar 90‐day mortality was observed in the two groups [21]. Although several key components of the glycocalyx were measured, other important constituents such as heparan sulfate and core proteoglycans were not, which may limit conclusions regarding overall glycocalyx shedding. Lastly, although a negative median difference of administered fluid (−606 mL), total fluid volume (−198 mL), and total fluid balance (−505 mL) was achieved in the restrictive fluid group compared to the standard fluid group at T1, it is possible that these fluid volumes were insufficient to produce a difference in glycocalyx shedding between groups. It is also worth noting that the volume of study‐assigned fluid administered was relatively small compared to the total fluid volume received, a common occurrence in the ICU setting due to “fluid creep”, which may have diluted any potential effect of the restrictive fluid strategy [29]. The fact that a positive median volume of total fluids given was recorded in the restrictive fluid group at timepoints 5 days and ICU discharge could perhaps be explained by the before‐mentioned higher incidence of GI infection in the restrictive fluid group, a condition that generally requires larger amounts of fluids to be administered and longer ICU stay.

## 5 Conclusion

In this underpowered sub‐study we found no statistically significant difference in endothelial glycocalyx shedding between adult ICU patients with septic shock randomized to restrictive versus standard IV fluid therapy in a subset of patients in the CLASSIC trial. Given the limited sample size and wide confidence intervals, the study was underpowered to detect small between‐group effects. We consider these findings hypothesis‐generating and further research is needed to confirm these results.

## Author Contributions

Morten Hylander Møller, Peter Buhl Hjortrup, Theis Lange, Tine Sylvest Meyhoff, Johan Mårtensson, Anders Perner, Eva Joelsson‐Alm and Maria Cronhjort contributed to the study conception and design. Data collection was carried out by Jens Christensen, Praleene Sivapalan, Sandra Jonmarker, Fredric Sjöberg, Anna Håkansson Gladh, Eva Joelsson‐Alm, Maria Cronhjort. Statistical analyses were performed by Hans Järnbert‐Pettersson and Jens Christensen. Jens Christensen drafted the first manuscript, and all authors contributed to the writing of the manuscript.

## Funding

This project was supported by The Swedish Society of Medicine (Grant number SLS‐934824), ALF Medicine Region Stockholm (FoUI‐955171) and a Clinical Researcher Grant from Region Stockholm RS 2021–0933.

## Disclosure

A detailed analysis plan for this sub‐study was published prior to data analysis [22]. This analysis plan differed from the initial ClinicalTrials.gov registration due to a subsequent decision to divide the original study into two separate studies.

## Ethics Statement

Ethics approval was acquired from the Swedish Ethical Review Authority (2018/1503–31 and 2019–01862). Written informed consent was obtained from all patients.

## Conflicts of Interest

The authors declare no conflicts of interest.

## Supporting information


**Table S1:** Intra assay CVs and LOQs.

**Table S2:** Fluid characteristics.

**Table S3:** Reporting checklist for randomised trial.

## Data Availability

The data that support the findings of this study are available on request from the corresponding author. The data are not publicly available due to privacy or ethical restrictions.

## Appendix A

### Best‐ and Worst‐Case Analysis

In the best‐case analysis mean levels of hyaluronan increased in both groups from T0 to T1. The increase was 41 ng/mL (95% CI 22–104) larger in the restrictive fluid group compared to the standard fluid group. The difference between groups at T0 was −51 ng/mL (95% CI 106–3) (restrictive fluid group 122 ng/mL (95% CI 84–159), standard fluid group 173 ng/mL (95% CI 133–213)) and at T1–10 ng/mL (95% CI 65–44) (restrictive fluid group 179 ng/mL (95% CI 142–216), standard fluid group 189 ng/mL (95% CI 149–229)).

An opposite trend was seen in the worst‐case analysis where mean levels of hyaluronan decreased in both groups from T0 to T1. The decrease was 49 ng/mL (95% CI 6–105) larger in the restrictive fluid group. The difference between groups at T0 was 61 ng/mL (95% CI 7–116) (restrictive fluid group 281 ng/mL (95% CI 244–318), standard fluid group 220 ng/mL (95% CI 180–260)) and at T1 12 ng/mL (95% CI 43–66) (restrictive fluid group 201 ng/mL (95% CI 164–238), standard fluid group 189 ng/mL (95% CI 149–229)).

### Multiple Imputations

In the analysis using multiple imputations mean levels of hyaluronan decreased by 1 ng/mL (95% CI 73–70) more in the standard fluid group compared to the restrictive fluid group from T0 to T1. The difference between groups at T0 was 1 ng/mL (95% CI 31–40) (restrictive fluid group 196 ng/mL (95% CI 134–259), standard fluid group 197 ng/mL (95% CI 154–241)) and at T1 0 ng/mL (95% CI 33–42) (restrictive fluid group 189 ng/mL (95% CI 152–226), standard fluid group 189 ng/mL (95% CI 151–227)).
