# Supplementary material for: Restrictive Versus Standard Intravenous Fluid Therapy and Endothelial Glycocalyx Shedding in ICU Patients With Septic Shock—A Preplanned Sub‐Study of the Randomized CLASSIC Trial
Source: Acta Anaesthesiol Scand. 2025 Dec 1;70(1):e70156. doi: 10.1111/aas.70156 (PMC12666609; doi:10.1111/aas.70156)
Supplement: Supplementary file 1 — Table S1: Intra assay CVs and LOQs. [file AAS-70-0-s003.docx]

# Supplementary Table 1. Intra assay CVs and LOQs

|  | **Intra-assay CV (%)** | **LLOQ** | **ULOQ** |
| --- | --- | --- | --- |
| Hyaluronan ng/mL | 3.6–7.2 % | 4.05 | 347.22 |
| TNFR1 ng/mL x10 | 4.3 % | 3.7 | 121.3 |
| Ang-2 ng/mL x10 | 3.8 % | 5.52 | 257.50 |
| MR-ProADM pg/mL | < 10 % | 3.62 | 250.22 |
| Syndecan-1 ng/mL x10 | 5.0 % | 9.1 | 288.2 |
| CD44 ng/mL x10 | 15.6 % | 7.5 | 237.5 |

*CV = Coefficient of Variation. LOQ = Limits of Quantification. LLOQ = Lower Limit of Quantification. ULOQ = Upper Limit of Quantification.*
